# Prediction of tuberculosis-specific mortality for older adult patients with pulmonary tuberculosis

**DOI:** 10.3389/fpubh.2024.1515867

**Published:** 2025-01-15

**Authors:** Sihua Wang, Ruohua Gu, Pengfei Ren, Yu Chen, Di Wu, Linlin Li

**Affiliations:** ^1^The Third People’s Hospital of Henan Province and Henan Hospital for Occupational Diseases, Zhengzhou, Henan Province, China; ^2^Department of Epidemiology and Health Statistics, College of Public Health, Zhengzhou University, Zhengzhou, Henan, China; ^3^Department of Tuberculosis, The Sixth People' s Hospital of Zhengzhou, Zhengzhou, Henan, China

**Keywords:** tuberculosis, competing risk model, TB-specific mortality, nomogram, older adult patients

## Abstract

**Background:**

This study aims to identify risk factors associated with tuberculosis-specific mortality (TSM) in older adult patients with pulmonary tuberculosis (TB) and to develop a competing risk nomogram for TSM prediction.

**Methods:**

We conducted a retrospective cohort study and randomly selected 528 older adult pulmonary TB patients hospitalized in designated hospitals in Henan Province between January 2015 and December 2020. The cumulative incidence function (CIF) was calculated for both TSM and non-tuberculosis-specific mortality (non-TSM). A Fine and Gray proportional subdistribution hazards model and a competing risk nomogram were developed to predict TSM in older adult patients.

**Results:**

The 5-year cumulative incidence functions (CIFs) for TSM and non-TSM were 9.7 and 9.4%, respectively. The Fine and Gray model identified advanced age, retreatment status, chest X-rays (CXR) cavities, and hypoalbuminemia as independent risk factors for TSM. The competing risk nomogram for TSM showed good calibration and excellent discriminative ability, achieving a concordance index (c-index) of 0.844 (95% confidence interval [CI]: 0.830–0.857).

**Conclusion:**

The Fine and Gray model provided an accurate evaluation of risk factors associated with TSM. The competing risk nomogram, developed using the Fine and Gray model, provided accurate and personalized predictions of TSM.

## Introduction

Tuberculosis (TB) is an infectious disease caused by *Mycobacterium tuberculosis*, primarily affecting the lungs (pulmonary TB). In 2022, TB ranked as the second leading cause of death from a single infectious agent globally, after COVID-19, causing nearly twice as many deaths as HIV/AIDS. In 2022, approximately 7.5 million new TB cases were reported worldwide, resulting in an estimated 1.30 million deaths (95% UI, 1.18–1.43 million) (1). TB poses a significant public health threat and ranks as the thirteenth leading cause of death globally. China, the country with the second-highest TB burden, faces challenges as TB increasingly affects older adults due to population aging, longer life expectancy and diseases reactivation (2). TB in the older adult (> 65 years) is a significant concern, not only because of its high prevalence but also due to its elevated mortality rate (3, 4). Research indicates that pulmonary TB patients aged ≥75 years have a higher mortality rate during treatment compared to younger patients (5). Advanced age is a recognized risk factor for TB development and poor patient prognosis, as highlighted by a recent systematic review (6). Therefore, addressing mortality risk factors and improving prediction models for older adult pulmonary TB patients is imperative.

Although numerous studies have investigated the prognosis of older adult pulmonary TB patients, most have emphasized overall survival through logistic or Cox regression analysis. Older adult individuals are prone to age-related fatal conditions, including pneumonia, multiple organ failure, and renal failure (7). Many older adult TB patients succumb to non-TB-related diseases, emphasizing the challenge of competing mortality risks in TB (8). In this context, Cox regression can overestimate or underestimate TB-specific mortality (TSM) risk due to its omission of competing risk events. Non-TB-specific mortality (non-TSM) should be accounted for as a competing risk when analyzing TSM. Thus, a competing risk model is advised when multiple mortality cause coexist (9). The Fine and Gray proportional subdistribution hazards model, a Competing risk methods, enhances the ability to discern risk factor effects on TSM. Recently, the nomogram has been recognized as a reliable and effective tool for predicting disease outcomes (10). Nomograms function by assigning scores to influencing factors based on their contributions in a regression model and calculating an individual’s total score to estimate outcomes (11).

In this study, we performed a competing risk analysis to calculate the cumulative incidence functions (CIFs) for tuberculosis-specific mortality (TSM) and non-tuberculosis-specific mortality (non-TSM) in older adult patients. In epidemiology, exposure is a key risk factor in assessing disease occurrence. Exposure refers to the behavior or environmental factor that puts an individual at risk of encountering potential pathogens or harmful substances. Therefore, we identified risk exposure factors for TSM and constructed a competing risk nomogram to predict TSM in older adult patients.

## Materials and methods

### Study population

**Figure 1:**
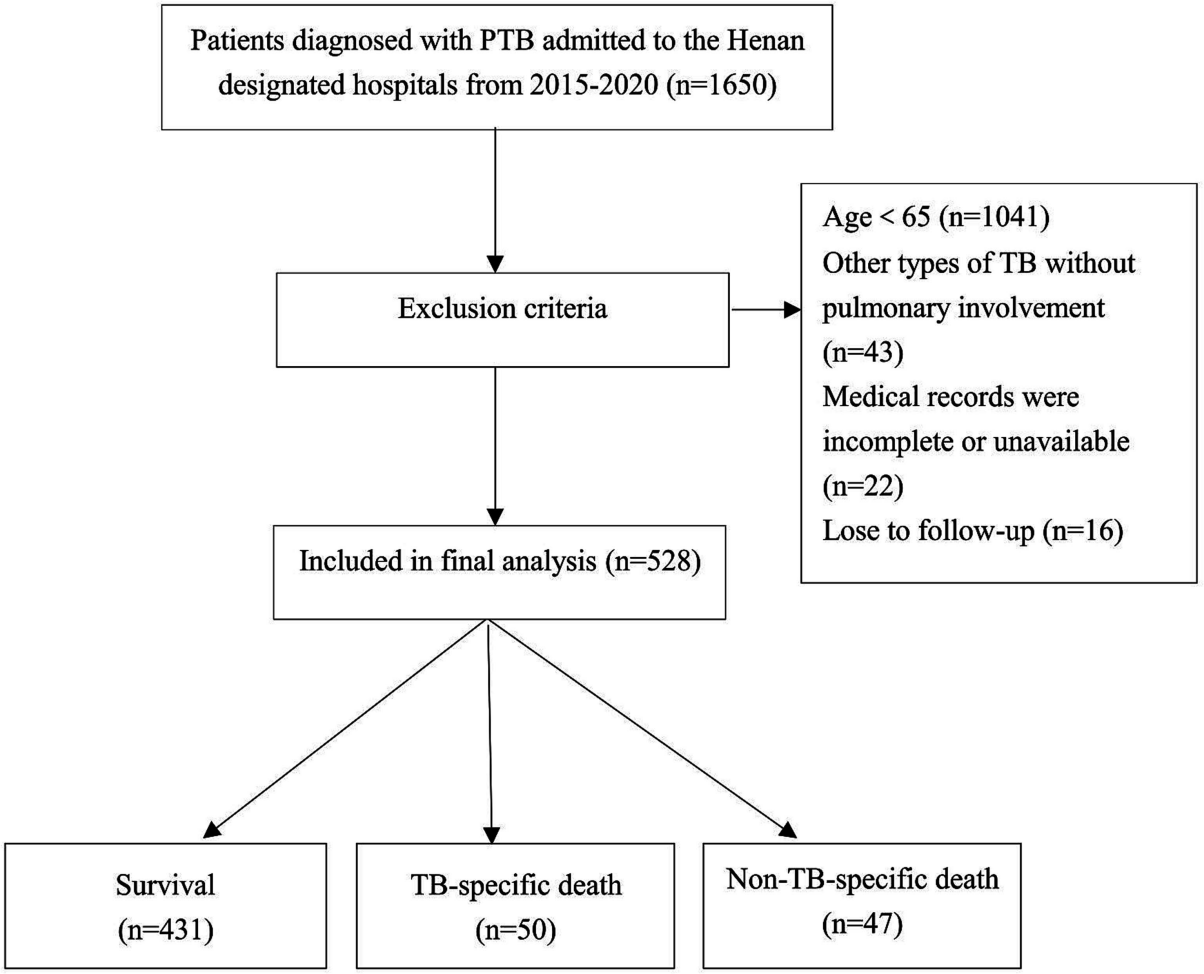
Flow-chart of participants.

This study randomly selected 14 out of 17 infectious disease hospitals in Henan Province as the study sites. Data were collected from 1,650 patients at these hospitals from January 2015 to December 2020. Patients were diagnosed with pulmonary TB according to the revised Chinese diagnostic criteria for tuberculosis (WS288-2017). Patients aged <65 years and those with miliary TB or other TB forms, such as TB pleuritis without pulmonary involvement, were excluded. Patients with incomplete or unavailable medical records or who did not complete follow-up were also excluded. After exclusions, 528 older adult patients remained and were included in the final analysis, as illustrated in Figure 1. The Institutional Ethics Committee approved the study and waived informed consent requirements due to its retrospective design.

### Data collection and follow-up

Demographic and clinical-pathological data were collected from medical records, including sex, age, body mass index (BMI), smoking status, alcohol use, comorbidities (diabetes mellitus, hypertension, malignancy), treatment category, baseline clinical details (sputum culture results, chest X-rays (CXR) findings), and laboratory results [serum albumin, absolute lymphocyte count (ALC) and C-reactive protein (CRP)], were obtained from medical records. Age groups were divided into 65–74 years, 75–84 years, and ≥ 85 years. BMI was categorized as underweight (<18.50 kg/m^2^), normal (18.50–23.99 kg/m^2^), and overweight or obese (≥24.00 kg/m^2^) according to Chinese reference standard (12). Hypoalbuminemia and lymphocytopenia were defined based on laboratory tests; serum albumin<35 g/L and ALC < 1.0 × 10^9^/L (13, 14). CRP levels were classified as high (≥ 10 mg/L) or normal (<10 mg/L). Information was collected from clinical records, outpatient visits, and follow-ups conducted via telephone or text messages. Follow-up outcomes were categorized into three groups: (1) Censored (survival); (2) Primary outcome (tuberculosis-specific death); and (3) Competing outcome (non-tuberculosis-specific death). The follow-up period was defined as the duration from hospital admission for pulmonary TB to the date of last contact or death.

### Definition of mortality

We used the definition from the Report of Verified Case of Tuberculosis by the US Centers for Disease Control and Prevention to differentiate between TB-specific and non-TB-specific deaths (15). TB-specific death was defined as death directly caused by TB (International Classification of Diseases 10th, ICD-10-CM code: A15–A19), including respiratory failure or severe hemoptysis due to tuberculosis. Non-TB-specific death was defined as any death not related to TB, determined by two respiratory specialists based on all available clinical data and death certification records.

### Statistical analysis

TSM and non-TSM were treated as competing events. The primary endpoint of the study was TSM. The cumulative incidence function (CIF) was used to estimate mortality. Subgroup analyses were also performed, and corresponding CIF curves were generated for each variable. Gray’s test was applied to assess differences in CIF values between subgroups. We used Fine and Gray’s proportional subdistribution hazards regression to construct the competing risk model. The Lasso regression model was employed to select predictors for the multivariate competing risk analysis. A competing risk nomogram based on Fine and Gray’s model was developed to predict TSM in older adult patients. The C-index was applied to evaluate discrimination, and a calibration plot was used to assess model calibration. Discrimination and calibration were assessed using R version 4.1.2 software.^1^ The R packages cmprsk, rms, mstate and glmnet were employed to build the model and nomogram, while the pec package was used to evaluate nomogram performance. All reported *p* values were two-sided, with *p* values <0.05 considered statistically significant.

## Results

### The incidences of mortality among older adult patients with pulmonary TB

The study population consisted of 528 older adult patients with pulmonary TB, as shown in Table 1. The median age at diagnosis was 73 years (range: 68–80 years). Of the patients, 55.9% were aged 65–74 years, 71.6% was male, and 74.4%received new treatment. Additionally, 40.2% were smokers, and 32.8% consumed alcohol. Regarding comorbidities, 118 patients (22.3%) had diabetes mellitus, 104 (19.7%) had hypertension, and 22 (4.2%) had malignancies. Of the patients, 46.6% tested positive for tuberculosis culture, and 45.1% had elevated CRP levels (≥10 mg/L). Hypoalbuminemia (serum albumin <35 g/L) was observed in 44.9% of patients, and lymphocytopenia (ALC <1.0 × 10^9^/L) in 29.9%. All patients underwent baseline CXR, with 24.8% showing evidence of cavitation.

**Table 1:** Five-year cumulative incidences of mortality among older adult patients with pulmonary TB.

Characteristic	*N* (%)	Event (%)	TB-specific mortality		Non-TB-specific mortality	
			5-year (%)	*p*	5-year (%)	*p*
Sex				0.304		0.457
Male	378 (71.59)	75 (77.32)	10.54		10.06	
Female	150 (28.41)	22 (22.68)	7.35		7.39	
Age (years)				0.003		<0.001
65–74	295 (55.87)	39 (40.21)	7.76		6.25	
75–84	186 (35.23)	36 (37.11)	9.14		10.84	
≥85	47 (8.90)	22 (22.68)	23.40		23.40	
BMI (kg/m^2^)				0.024		0.422
<18.50	105 (19.89)	30 (30.93)	16.23		12.82	
18.50–23.99	293 (55.49)	44 (45.36)	7.42		7.85	
≥24.00	130 (24.62)	23 (23.71)	9.40		10.15	
Smoking				0.524		0.218
No	316 (59.85)	64 (65.98)	10.19		10.79	
Yes	212 (40.15)	33 (34.02)	8.79		7.33	
Alcohol use				0.894		0.131
No	355 (67.23)	69 (71.13)	9.39		10.80	
Yes	173 (32.77)	28 (28.87)	10.03		6.56	
Treatment category				<0.001		0.474
New	393 (74.43)	58 (59.79)	6.41		8.75	
Retreatment	135 (25.57)	39 (40.21)	19.02		11.04	
DM				0.305		0.112
No	410 (77.65)	68 (70.10)	9.01		8.19	
Yes	118 (22.35)	29 (29.90)	11.99		13.28	
Hypertension				0.629		0.233
No	424 (80.30)	74 (76.29)	9.37		8.67	
Yes	104 (19.70)	23 (23.71)	10.76		12.10	
Malignancy				0.440		0.999
No	506 (95.83)	94 (96.91)	9.88		9.14	
Yes	22 (4.17)	3 (3.09)	4.55		21.07	
TB Culture				0.079		0.873
Negative	282 (53.41)	46 (47.42)	7.67		9.22	
Positive	246 (46.59)	51 (52.58)	11.85		9.54	
Cavities on CXR				<0.001		0.904
No	397 (75.19)	53 (54.64)	4.57		9.07	
Yes	131 (24.81)	44 (45.36)	25.41		10.86	
Hypoalbuminemia				<0.001		0.105
No	291 (55.11)	34 (35.05)	4.47		7.81	
Yes	237 (44.89)	63 (64.95)	16.12		11.15	
Lymphocytopenia				0.052		0.498
No	370 (70.08)	68 (70.10)	10.05		8.73	
Yes	158 (29.92)	29 (29.90)	8.78		10.87	
CRP (mg/L)				0.002		<0.001
<10	290 (54.92)	32 (32.99)	6.00		5.49	
≥10	238 (45.08)	65 (67.01)	14.01		14.25	

BMI, body mass index; CXR, chest X-rays; CRP, C-reactive protein.

**Figure 2:**
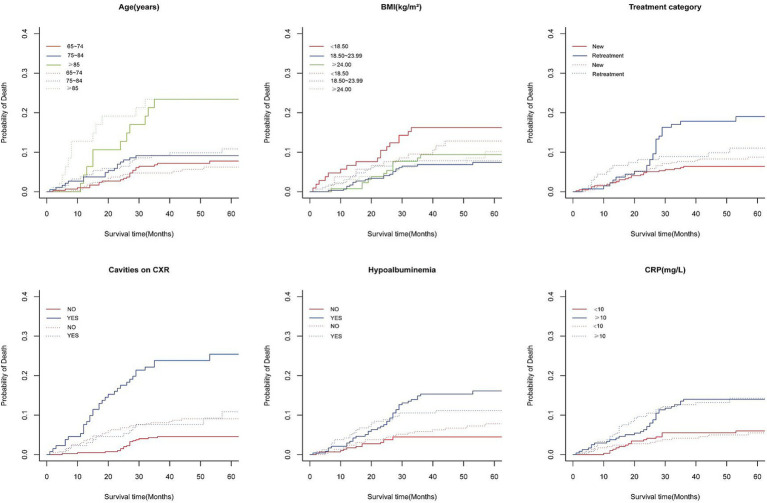
Cumulative incidence curves of deaths according to patient characteristics (solid line represents TB-specific death; dotted line represents Non-TB-specific death).

The median follow-up duration was 47 months, with a range of 36 to 61 months. At the end of follow-up period, 431 patients (81.6%) were alive, while 97 patients (18.4%) had died, including 50 tuberculosis-specific deaths (9.5%) and 47 non-tuberculosis-specific deaths (8.9%). The 5-year cumulative incidence function (CIF) estimates for TSM and non-TSM based on patient characteristics, are shown in Table 1. The 5-year CIF for TSM was 9.7%, and for non-TSM, it was 9.4%. The competing risk model analysis indicated a higher cumulative incidence of TSM in patients with advanced age (*p* = 0.003), underweight (*p* = 0.024), retreatment (*p* < 0.001), cavities on CXR (*p* < 0.001), hypoalbuminemia (*p* < 0.001) and CRP ≥ 10 mg/L (*p* = 0.002). Advanced age (*p* < 0.001) and CRP ≥ 10 mg/L (*p* < 0.001) were significantly associated with an increased cumulative incidence of non-TSM. Patient characteristics with significant differences in the CIF for TSM are shown in Figure 2.

### Variable selection by Lasso regression

**Figure 3:**
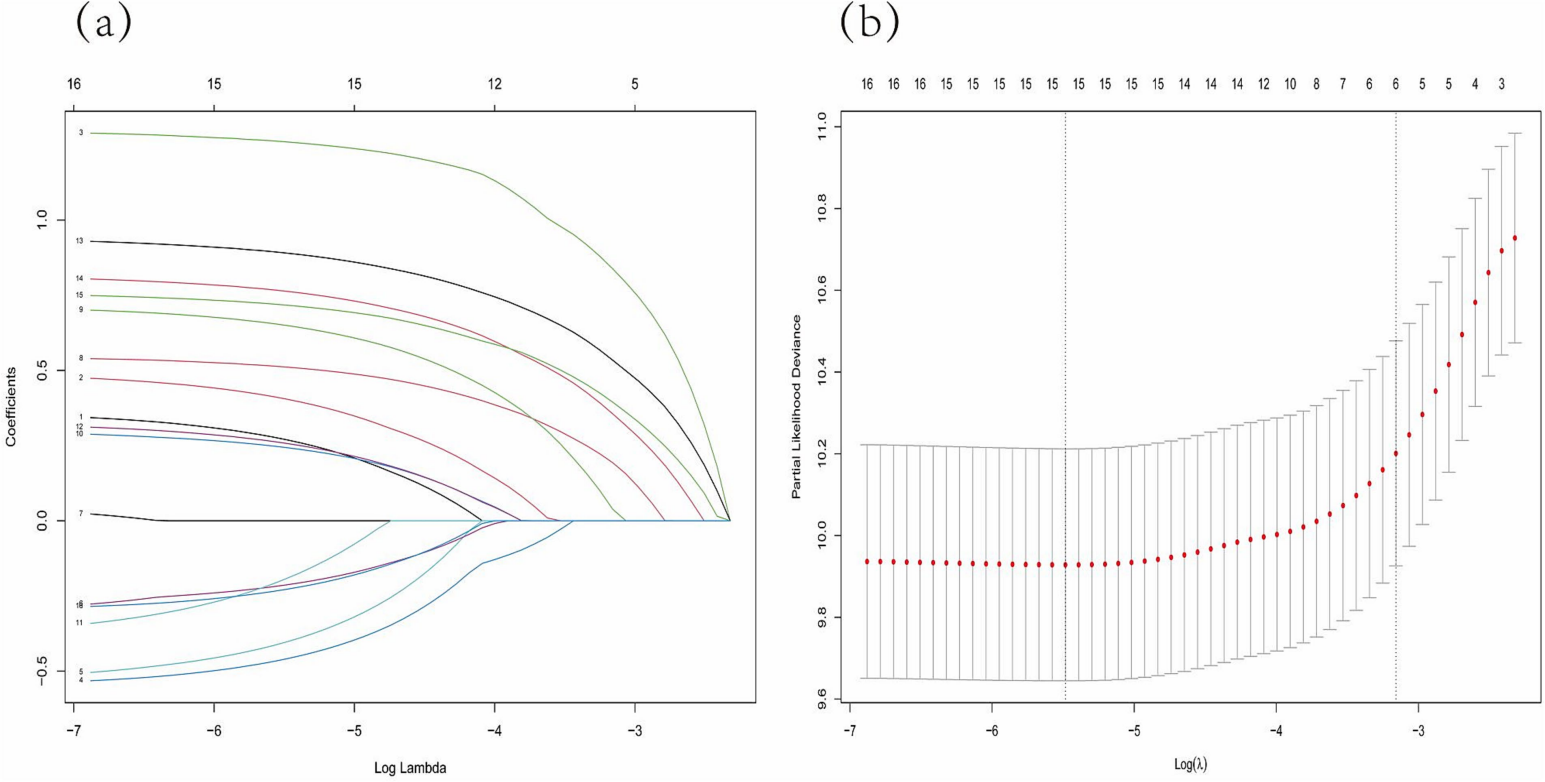
Lasso regression model was adopted to determine predictive variables for the further multivariate analysis. **(A)** LASSO coefficient patterns of the features. **(B)** Ten-time cross-verification for tuning parameter selection in the Lasso model.

After converting the 14 potential influencing factors into dummy variables, 16 candidate variables were included in the Lasso regression for selection. In Figure 3A, each curve represents the change in coefficient for each candidate variable. Figure 3B illustrates the process of selecting the optimal *λ* value through 10-fold cross-validation. The optimal λ value was chosen when the error reached λ_min_ + 1 standard error. Five predictor variables were identified: age (*β* = 0.792), treatment category (*β* = 0.156), cavities on CXR (*β* = 0.499), hypoalbuminemia (*β* = 0.317), and CRP level (*β* = 0.362).

### Analysis of influencing factors of tubercular specific death based on Fine and Gray proportional hazards model

The predictors identified by Lasso regression were included in the multivariate competing risk regression analysis. The Fine and Gray proportional subdistribution hazards model for Tuberculosis-specific mortality (TSM) in older adult patients is presented in Table 2. Patients aged ≥85 years (HR 2.96; 95% CI: 1.52–5.77, *p* = 0.002) had a significantly higher risk of TSM compared to those aged 65–74 years. Retreatment patients (HR 2.53; 95% CI: 1.45–4.42, *p* = 0.001), patients with hypoalbuminemia (HR 3.17; 95% CI: 1.65–6.08, *p* < 0.001), and cavities on chest X-ray (CXR) (HR 5.11; 95% CI: 2.79–9.36, *p* < 0.001) were associated with an increased risk of TSM. Advanced age and elevated CRP levels were significant predictors of non-TSM. Patients aged ≥85 years (HR 4.48; 95% CI: 2.04–9.88, *p* < 0.001) had a significantly higher risk of non-TSM compared to those aged 65–74 years. Additionally, patients with CRP levels ≥10 mg/L had an increased risk of non-TSM (HR 2.67; 95% CI: 1.43–5.00, *p* = 0.002).

**Table 2:** Fine and Gray proportional subdistribution hazards model of probabilities of mortality for older adult patients with pulmonary TB.

Characteristic	TB-specific mortality			Non-TB-specific mortality		
	HR	95% CI	*p*	HR	95% CI	*p*
Age (years)						
65–74	Ref	—	—	Ref	—	—
75–84	1.45	0.76–2.76	0.260	1.83	0.95–3.53	0.072
≥85	2.96	1.52–5.77	0.002	4.48	2.04–9.88	<0.001
Treatment category						
New	Ref	—	—	Ref	—	—
Retreatment	2.53	1.45–4.42	0.001	1.25	0.65–2.39	0.500
Cavities on CXR						
No	Ref	—	—	Ref		
Yes	5.11	2.79–9.36	<0.001	0.79	0.39–1.57	0.490
Hypoalbuminemia						
No	Ref	—	—	Ref	—	—
Yes	3.17	1.65–6.08	<0.001	1.38	0.77–2.46	0.280
CRP (mg/L)						
<10	Ref	—	—	Ref	—	—
≥10	1.57	0.85–2.88	0.150	2.67	1.43–5.00	0.002

HR, hazard ratio; CI, confidence interval; Ref, reference.

### Construction of prediction model nomogram and evaluation of prediction effect

**Figure 4:**
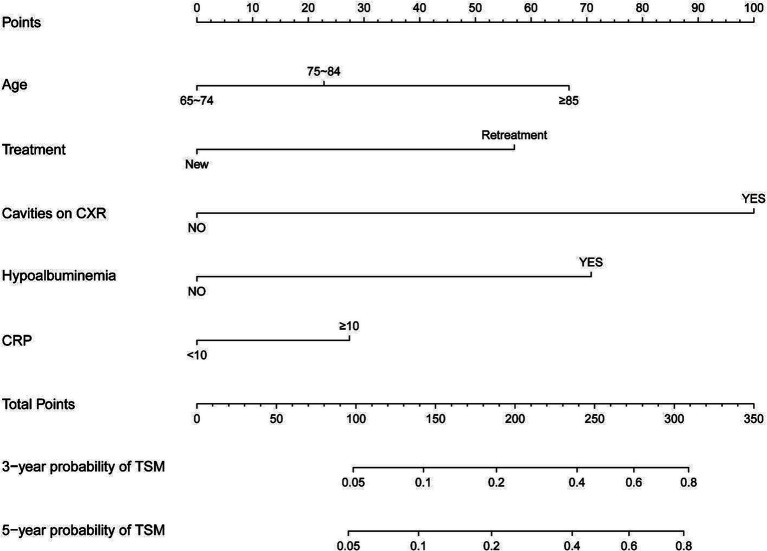
Nomogram for predicting 3- and 5-year probabilities of TSM in older adult patients with pulmonary tuberculosis. TSM, tuberculosis-specific mortality.

**Figure 5:**
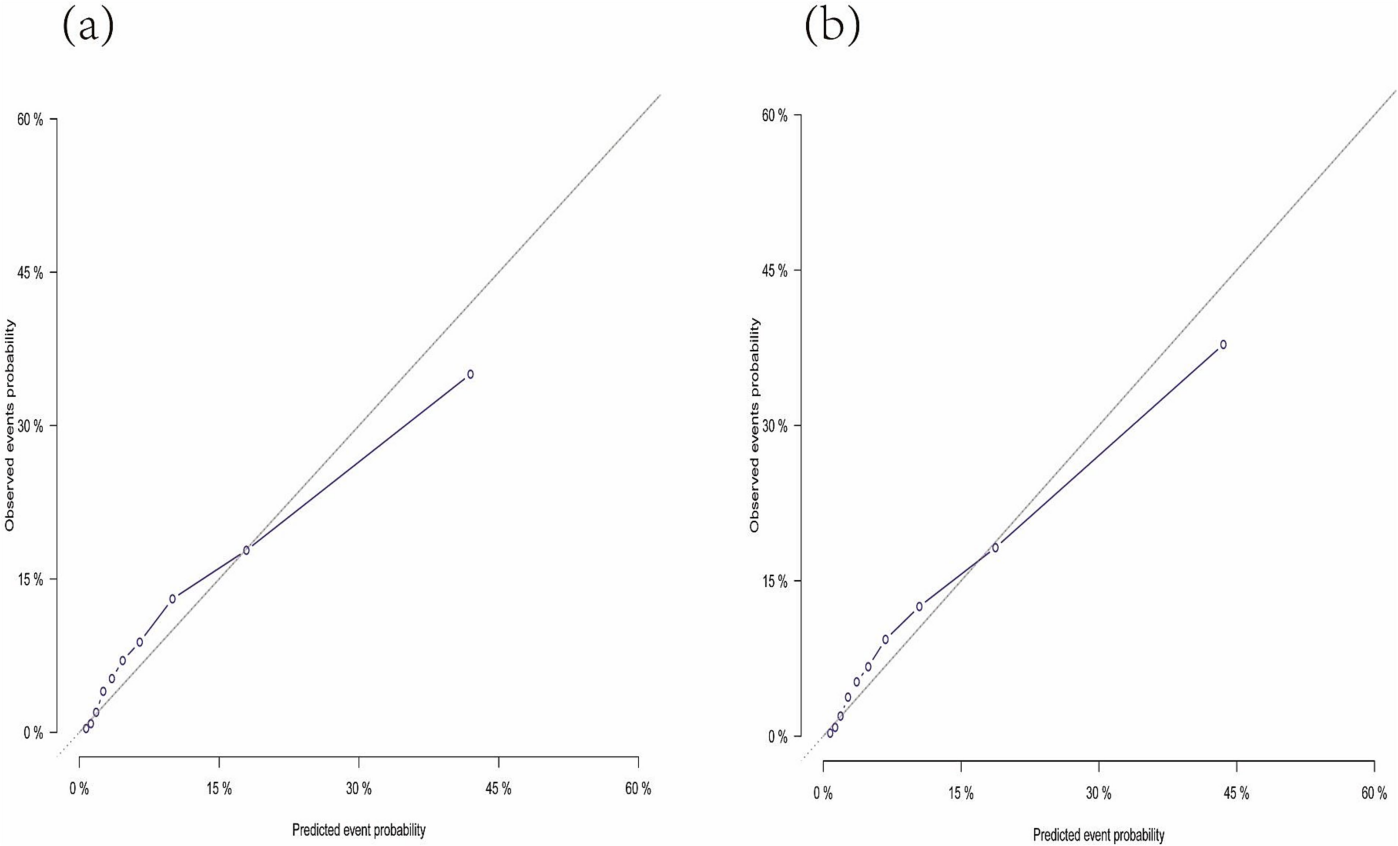
Calibration plot indicating the nomogram. **(A)** The calibration curve predicting 3-year tuberculosis-specific mortality. **(B)** The calibration curve predicting 5-year tuberculosis-specific mortality.

Figure 4 presents the nomogram developed using the Fine and Gray proportional subdistribution hazards model. This nomogram predicts the 3- and 5-year TSM probabilities for older adult pulmonary TB patients by summing points based on individual characteristics. A straight line is drawn from the total score to the bottom of the graph to estimate the TSM probability. The model demonstrated strong discriminative ability, with a C-index of 0.844 (95% CI: 0.830–0.857). The calibration plot of the CIF is presented in Figure 5. Points close to the 45-degree line indicate strong agreement between the predicted probabilities and actual outcomes.

## Discussion

This retrospective study evaluates the cumulative incidence of mortality in older adult pulmonary TB patients hospitalized in Henan from 2015 to 2020. This study found that advanced age, retreatment, cavities on CXR, and hypoalbuminemia were significantly associated with a higher cumulative incidence of TSM, as determined by the Fine and Gray proportional subdistribution hazards model. Furthermore, the nomogram based on the Fine and Gray model demonstrated strong discriminative and calibration abilities, as evidenced by the C-index and calibration plot. To our knowledge, this is the first the competitive risk nomogram developed to provide precise, personalized predictions of TSM.

Numerous studies have demonstrated poor clinical outcomes and prognosis in older adult patients with pulmonary TB (1, 16–18). The present study found that the five-year cumulative incidence of overall mortality was 19.1%. This rate is comparable to the findings of the studies in Colombia (21.8%) (19) and Japan (20.7%) (20). However, it was higher than the rate reported in Italy (13.0%) (21), but lower than that in Taiwan (32.7%) (8). The mortality rate in older adult patients in this study was generally higher or comparable to that in some developed countries. These differences may reflect variations in healthcare quality and genetic factors across regions. In our study, the five-year cumulative incidences of TSM and non-TSM were 9.7 and 9.4%, respectively. Among all deaths, 49.2% were due to causes other than tuberculosis. These findings emphasize the importance of considering non-TSM as a competing risk in older adult patients, who are at greater risk of severe comorbidities and mortality from other causes.

In our competing risk analysis, advanced age emerged as a significant predictor of TSM and non-TSM. Consistent with a systematic review (6), age independently influenced the prognosis of older adult patients with TB. Hase (5) and Murali (22) found that advanced age was linked to a higher risk of TB-related mortality. Lin et al. (23) reported adjusted odds ratio (aOR) of 1.87 and 3.40 for mortality in patients aged 75–84 and ≥ 85 years, respectively, compared to those aged 65–74 years. Increased mortality among older patients with pulmonary TB may result from declining immunity and age-related comorbidities, such as immunosuppression, anemia, and adverse drug reactions (24, 25). Additionally, older adult TB patients were more likely to experience delays in diagnosis and treatment (26), which may potentially contribute to their higher mortality.

Our study found that retreatment significantly increases the risk of TSM in older adult patients, consistent with previous studies (27, 28). Ananthakrishnan et al. (29) reported similar findings, with retreatment patients having a higher risk of unfavorable outcomes (OR 2.5; 95% CI: 1.9–3.2) in the older adult. Higher mortality in retreatment cases may result from greater non-compliance with TB treatment, leading to drug-resistant TB (28). The prognostic role of cavities on CXR in older adult patients is still controversial. Studies by Di Gennaro (21) and Wu (30) identified cavities on CXR as a risk factor for TB-specific deaths in older adult patients, which aligns with our findings. In contrast, Yen et al. (8) suggested that cavities on chest radiographs were associated with a lower risk of mortality. The protective effect may be attributed to reduced diagnostic delays associated with cavitary lesions. Honjo et al. (31) found in a retrospective study that cavities on CXR did not affect TB-related mortality when analyzed using the competing risk regression model. Therefore, future studies should include larger samples and longer follow-up periods to better investigate the prognostic role of cavities on CXR in older adult patients with pulmonary TB.

Our study identified hypoalbuminemia as an independent risk factor for TSM. However, most studies have evaluated serum albumin levels rather than hypoalbuminemia as a predictor of mortality in older adult patients. The association between low albumin levels and poor prognosis aligns with previous studies (16, 31). Tanaka et al. (32) similarly found that albumin levels were significantly associated with in-hospital mortality in older adult patients after adjusting for confounders. Low serum albumin levels in older adult patients may reflect malnutrition and severe inflammation caused by TB infection. However, clinical evidence supporting the efficacy of nutritional intervention in patients with pulmonary TB remains lacking. CRP, an acute-phase protein, serves as an inflammatory marker by enhancing cell-mediated immunity through phagocytosis, promoting chemotaxis, and activating platelets (33). Elevated CRP levels may indicate the pulmonary TB severity, as they are associated with prolonged sputum conversion time in severe cases (34). Nakamura et al. (35) found in a retrospective cohort study that CRP levels were independently associated with overall mortality using the multivariate Cox model. However, studies using the Cox model may overestimate TSM incidence. In our competing risk analysis, high CRP levels (≥10 mg/L), indicating severe inflammation, were an independent predictor of non-TSM but not TSM.

In this study, the five-year cumulative incidences of non-TSM and TSM were comparable, at 9.4 and 9.7%, respectively. Thus, competing causes of mortality play a crucial role in prognosis evaluation. In contrast to traditional Cox regression, which treats competing events as censored data and may introduce bias, the Fine and Gray proportional subdistribution hazards model effectively manages survival data with multiple potential endpoints and directly estimates the impact of each covariate on TSM. This model has been recently applied to chronic diseases, including coronary heart disease, stroke, and common cancers. To date, a single international study (31) has utilized the Fine and Gray model to investigate the prognosis of older adult TB patients. In this study, we constructed a competing risk nomogram to predict 3- and 5-year TSM. The nomogram, incorporating demographic and clinicopathological characteristics, demonstrated excellent calibrated and considerable predictive efficiency.

This study has several potential limitations: First, it included older adult patients with pulmonary TB who were hospitalized in designated hospitals from 2015 to 2020. Approximately 12% of the patients were diagnosed between 2018 and 2020, resulting in a relatively short follow-up period. Extending follow-up period could improve the model’s predictive accuracy. Second, as a retrospective cohort study, this research cannot establish causation and may be influenced by selection bias and unmeasured confounding factors, even with the use of competing risk regression models. Additionally, the model did not account for several treatment-related factors, such as treatment regimen, sputum culture or smear conversion time, and treatment delays. These factors may affect the patient prognosis. Finally, this study lacked external validation in independent populations. The applicability of the nomogram to other regions or ethnic groups requires further validation through multicenter studies. Therefore, the universal applicability of this model requires verification, which could influence its clinical utility.

## Conclusion

This study identified age, treatment category, cavities on chest X-ray (CXR), and hypoalbuminemia as independent predictors of TSM through the Fine and Gray proportional subdistribution hazards model. A competing risk nomogram was developed based on this model to estimate 3- and 5-year TSM probabilities. The nomogram exhibited strong discrimination and calibration, as evidenced by the C-index and calibration plot. This well-calibrated nomogram can support clinical decision-making by delivering prognostic estimates and tailored treatment recommendations for older adult patients with pulmonary TB.

## Data Availability

The raw data supporting the conclusions of this article will be made available by the authors, without undue reservation.

## Footnotes

^1^ https://www.r-project.org
